# Long-term bone and lung consequences associated with hospital-acquired severe acute respiratory syndrome: a 15-year follow-up from a prospective cohort study

**DOI:** 10.1038/s41413-020-0084-5

**Published:** 2020-02-14

**Authors:** Peixun Zhang, Jia Li, Huixin Liu, Na Han, Jiabao Ju, Yuhui Kou, Lei Chen, Mengxi Jiang, Feng Pan, Yali Zheng, Zhancheng Gao, Baoguo Jiang

**Affiliations:** 1 Department of Orthopedics and Trauma, Peking University People’s Hospital, Beijing, China; 2 Department of Respiratory Medicine, Peking University People’s Hospital, Beijing, China; 3 Department of Clinical Epidemiology and Biostatistics, Peking University People’s Hospital, Beijing, China; 4 Department of Central Laboratory, Peking University People’s Hospital, Beijing, China; 5 Department of Education, Peking University People’s Hospital, Beijing, China; 6 Department of Radiology, Peking University People’s Hospital, Beijing, China

**Keywords:** Calcium and phosphate metabolic disorders, Bone

## Abstract

The most severe sequelae after rehabilitation from SARS are femoral head necrosis and pulmonary fibrosis. We performed a 15-year follow-up on the lung and bone conditions of SARS patients. We evaluated the recovery from lung damage and femoral head necrosis in an observational cohort study of SARS patients using pulmonary CT scans, hip joint MRI examinations, pulmonary function tests and hip joint function questionnaires. Eighty medical staff contracted SARS in 2003. Two patients died of SARS, and 78 were enrolled in this study from August 2003 to March 2018. Seventy-one patients completed the 15-year follow-up. The percentage of pulmonary lesions on CT scans diminished from 2003 (9.40 ± 7.83)% to 2004 (3.20 ± 4.78)% (*P* < 0.001) and remained stable thereafter until 2018 (4.60 ± 6.37)%. Between 2006 and 2018, the proportion of patients with interstitial changes who had improved pulmonary function was lower than that of patients without lesions, as demonstrated by the one-second ratio (FEV_1_/FVC%, *t* = 2.21, *P* = 0.04) and mid-flow of maximum expiration (FEF_25%–75%_, *t* = 2.76, *P* = 0.01). The volume of femoral head necrosis decreased significantly from 2003 (38.83 ± 21.01)% to 2005 (30.38 ± 20.23)% (*P* = 0.000 2), then declined slowly from 2005 to 2013 (28.99 ± 20.59)% and plateaued until 2018 (25.52 ± 15.51)%. Pulmonary interstitial damage and functional decline caused by SARS mostly recovered, with a greater extent of recovery within 2 years after rehabilitation. Femoral head necrosis induced by large doses of steroid pulse therapy in SARS patients was not progressive and was partially reversible.

## Introduction

The sudden outbreak of the severe acute respiratory syndrome (SARS) virus in early 2003 disturbed the world, especially China.^1–3^ Physicians and microbiologists have made great achievements in understanding the pathogen and effectively controlling its spread. The World Health Organization identified the source of the disease as SARS-CoV coronavirus on April 16, 2003.^4–6^ A total of 5327 individuals were diagnosed with SARS in China, and 349 patients died.^7^ In Beijing, SARS first broke out in Peking University People’s Hospital, where 80 medical staff contracted the virus, two of whom died subsequently. This cohort became the largest patient population worldwide and was composed of healthcare workers infected during their employment. Under the guidance on managing infectious diseases, Peking University People’s Hospital was isolated by the government for 22 days. The main clinical manifestation after infection was high fever and severe lung inflammation.^5,6^ The patients who survived had residual pulmonary fibrosis as well as osteonecrosis resulting from treatment with large doses of steroid pulse therapy. Several studies have investigated the long-term outcomes of recovered SARS patients, especially with respect to lung and bone damage, but no follow-up periods of more than 7 years have been reported. We conducted a comprehensive 15-year follow-up of healthcare workers with nosocomial SARS to evaluate their health utility after rehabilitation and obtain a new understanding of the associated pulmonary damage and femoral head necrosis.

## Results

**Fig. 1:**
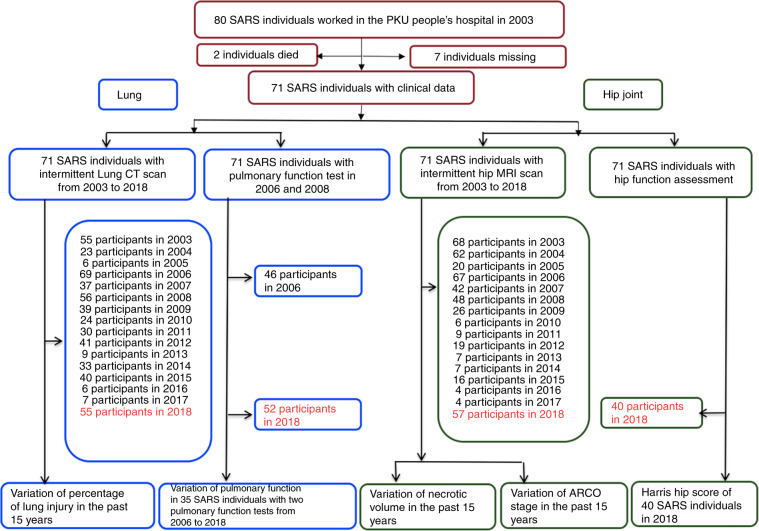
Flow diagram of the follow-up examinations

Eighty health care providers at Peking University People’s Hospital contracted SARS in 2003. Two patients died of SARS in 2003, and seven patients declined to participate in the study. A total of 71 patients (57 female) completed a 15-year follow-up from 2003 to 2018 (Fig. 1).

We surveyed the length of hospital stay and treatment regimes in 2018 and evaluated health utility via questionnaires. The results showed that the length of hospital stay ranged from 10 to 70 days in 2003, with a median of 33 days (30, 42.5), and daily methylprednisolone doses ranged from 0 to 800 mg, with a median of 160 mg per day (100, 400). Only 38 patients remembered their cumulative steroid dosage (methylprednisolone via intravenous injection) ranging from 0 to 20 000 mg with a median of 3 450 mg (2 285, 5 225 mg). A total of 53 out of 71 patients were still employed in 2018, nine were retired, and nine were on long-term sick leave. The enrolled patients had various comorbidities; three patients were complicated with anxiety and depression requiring treatment, sixteen had hypertension, five had diabetes, seven had peptic ulcers, and one had Sjogren’s syndrome. The characteristics of the patients are shown in Table 1.

**Table 1:** Characteristics of the 71 SARS patients included in the final analysis in 2003

Characteristics	Categories	*N* (%)
General status		
Age (*n* = 71)		
	<30	30 (42.25%)
	30–39	28 (39.44%)
	40–49	8 (11.27%)
	≥50	5 (7.04%)
Gender (*n* = 71)		
	Female	56 (78.87%)
	Male	15 (21.13%)
Personal medical history (*n* = 58)		
	Hypertension	4 (6.90%)
	Diabetes	1 (1.72%)
	Sjogren’s syndrome	1 (1.72%)
	Smoke	3 (5.17%)
Clinical symptoms		
Fever (*n* = 56)	Yes	56 (100%)
Rigor (*n* = 57)	Yes	36 (63.16%)
Dyspnea (*n* = 57)	Yes	35 (61.40%)
Myalgia (*n* = 56)	Yes	37 (66.07%)
Cough (*n* = 57)	Yes	29 (50.88%)
Expectoration (*n* = 56)	Yes	10 (17.86%)
Diarrhea (*n* = 56)	Yes	10 (17.86%)
Anorexia (*n* = 56)	Yes	23 (41.07%)
Malaise (*n* = 56)	Yes	42 (75.00%)
Treatment		
Ribavirin (*n* = 52)	Used	46 (88.46%)
MP (*n* = 58)	Used	56 (96.55%)
Non-invasive ventilation (*n* = 45)	Used	14 (31.11%)
Invasive ventilation (*n* = 58)	Used	1 (1.72%)
Severity		
Lung injury (*n* = 71)	Yes	27 (38.03%)
Bone injury (*n* = 71)	Yes	15 (21.13%)

We performed pulmonary function tests in 2006 and 2018. The outcomes in 2006 revealed that 10 out of 46 (21.74%) patients had restrictive ventilation dysfunction. Sixteen out of 46 (34.78%) patients had reduced diffusion capacity with an ~70%–80% predicted value, indicating a mild reduction. In 2018, 15 years after being infected, we reperformed pulmonary function tests for the cohort. One out of 52 patients (1.92%) had obstructive ventilation dysfunction, while none had restrictive ventilation dysfunction. However, the number of patients with impaired FEF_25%–75%_ values increased (16/52, 40.38%). Eighteen patients (38.46%) had reduced diffusion capacity, indicating that the ratio slightly increased, but the difference was not statistically significant.

A total of 35 patients underwent pulmonary functional tests in both 2006 and 2018. The cohort was further divided into two groups (normal CT findings and abnormal CT findings group) based on their CT findings in 2003, 6 months after being infected. The pulmonary function test parameters were compared between the normal CT findings group (*N* = 22) and the abnormal CT findings group (*N* = 13). The results are shown in Table 2, and there were no substantial changes in pulmonary function in the abnormal CT finding group in the past few years. Between the two groups, only the forced expiratory volume in 1 s/forced vital capacity (FEV_1_/FVC ratio) and the FEF_25%–75%_ value were significantly reduced (*P* = 0.04 and *P* = 0.01, respectively). The other parameters, including total lung capacity (TLC) and carbon monoxide diffusing capacity of the lungs (DLCO), did not significantly change 15 years after the initial infection. The pulmonary function in 2018 was better than that in 2006 for patients whose CT scans showed no abnormalities after recovery in 2003.

**Table 2:** Comparison of pulmonary function items in 2006 and 2018 in SARS patients with or without a pulmonary CT abnormity after recovery in 2003

Characteristics	Abnormal CT (*n* = 13)			Normal CT (*n* = 22)			*t*	*P*
	2006	2018	2018–2006	2006	2018	2018–2006		
FVC/L	3.57 ± 1.02	3.36 ± 0.98	−0.20 ± 0.34	3.59 ± 0.67	3.43 ± 0.52	−0.15 ± 0.36	0.38	0.703 0
FVC of predicted/%	102.05 ± 19.57	104.54 ± 16.06	2.49 ± 11.16	97.31 ± 14.00	102.53 ± 14.27	5.22 ± 9.29	0.78	0.441 8
FEV_1_/L	2.98 ± 0.79	2.69 ± 0.74	−0.29 ± 0.27	3.05 ± 0.57	2.86 ± 0.45	−0.20 ± 0.30	0.93	0.357 8
FEV_1_ of predicted/%	101.37 ± 17.85	99.75 ± 16.92	−1.62 ± 8.10	96.18 ± 13.06	100.23 ± 13.41	4.05 ± 8.56	1.93	0.062 1
(FEV_1_/FVC)/%	84.36 ± 6.07	79.32 ± 5.27	−5.04 ± 4.04	85.25 ± 6.31	83.26 ± 4.34	−2.00 ± 4.13	2.12	0.041 4
FEF_25%−75%_	86.49 ± 19.50	71.16 ± 20.39	−15.33 ± 16.49	84.62 ± 22.98	83.88 ± 23.19	−0.75 ± 14.27	2.76	0.009 4
DLCO/(mmol·min^−1^ per kPa)	7.85 ± 2.07	7.39 ± 1.92	−0.46 ± 1.27	7.67 ± 1.51	7.18 ± 1.35	−0.49 ± 1.10	−0.07	0.942 4
DLCOc of predicted/%	87.81 ± 11.25	88.22 ± 12.67	0.40 ± 13.85	81.65 ± 9.02	81.99 ± 11.82	0.35 ± 11.17	−0.01	0.988 5
DLCO/VA of predicted/%	98.70 ± 10.05	99.00 ± 15.45	0.30 ± 11.63	92.45 ± 12.10	88.53 ± 16.32	−3.92 ± 10.80	−1.09	0.285 2
TLC/L	5.22 ± 1.32	5.49 ± 1.22	0.27 ± 0.64	5.24 ± 0.69	5.34 ± 0.59	0.10 ± 0.67	−0.73	0.471 0
TLC of predicted/%	97.62 ± 16.56	103.12 ± 11.28	5.51 ± 13.87	98.68 ± 10.07	102.22 ± 10.41	3.54 ± 11.92	−0.45	0.659 2
(RV/TLC)/%	35.26 ± 8.69	39.15 ± 7.38	3.89 ± 8.35	32.85 ± 7.07	38.24 ± 5.60	5.39 ± 6.61	0.59	0.560 9
RV/TLC of predicted/%	106.75 ± 19.57	106.43 ± 14.27	−0.32 ± 25.13	109.66 ± 18.83	113.90 ± 17.00	4.24 ± 21.07	0.58	0.569 1

**Fig. 2:**
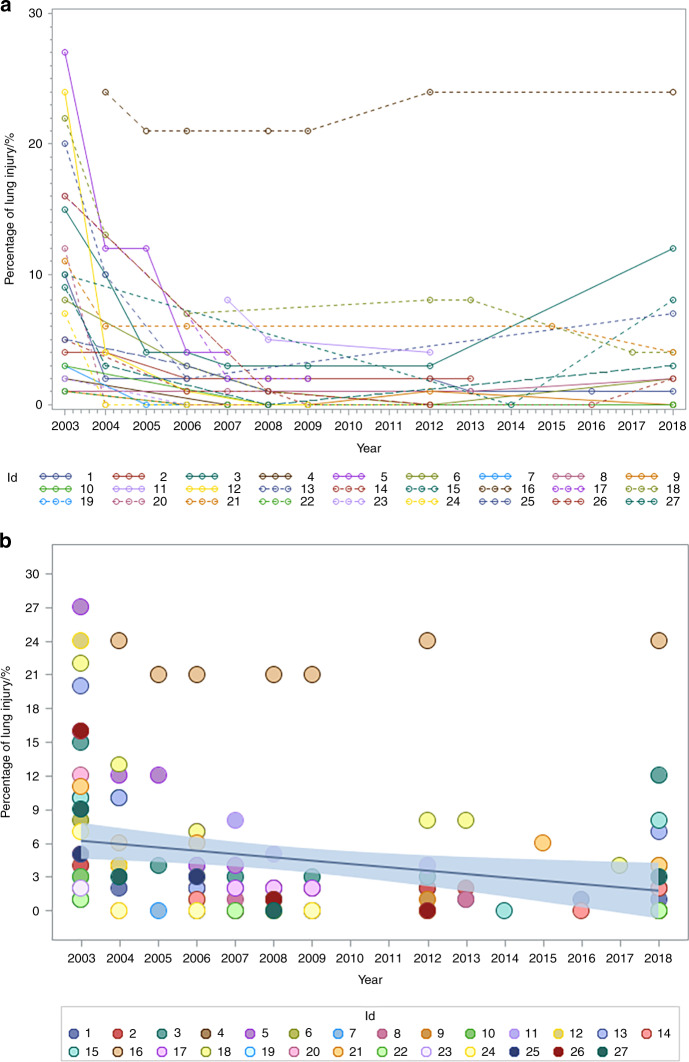
Trends in percentage change of lesion area on pulmonary CT scan. **a** Changes in percentage in the injured lung CT. **b** Bubble plots and linear regression of changes in the percentage of injured lung on CT

The pulmonary CT scans of the 71 SARS patients showed that 27 patients exhibited ground-glass opacities or cord-like consolidations during the 15-year follow-up. Two patients had no abnormalities on their pulmonary CT scans in 2003, but ground-glass-like changes were observed in both 2004 and 2007. We calculated the percentage of area with lesions on two sections of the CT scan and analyzed the overall variations in the proportion of lung area with lesions in 27 patients (Fig. 2a, b).

The linear regression showed that in the 27 patients who underwent pulmonary CT scans from 2003 to 2018, the percentage of lesions gradually decreased (*t* = −2.56, *P* = 0.01). The mixed-model showed that the proportion of lesions differed every year during the follow-up period (*F* = 7.79, *P* < 0.001), with the proportion in 2004 being lower than that in 2003, and lower in 2013 than in 2012. No differences were observed between the last two remaining years. The results showed that the incidence of pulmonary lesions in SARS patients decreased annually. Lesion absorption and recovery occurred to a greater extent between 2003 and 2004 and then remained stable until 2018.

Fifteen out of 71 patients were confirmed to have femoral head necrosis by hip joint MRI in 2003. In the cohort, seven patients had unilateral osteonecrosis, and eight had bilateral osteonecrosis. The total number of patients with femoral head necrosis was 23, as some patients had bilateral lesions.

**Fig. 3:**
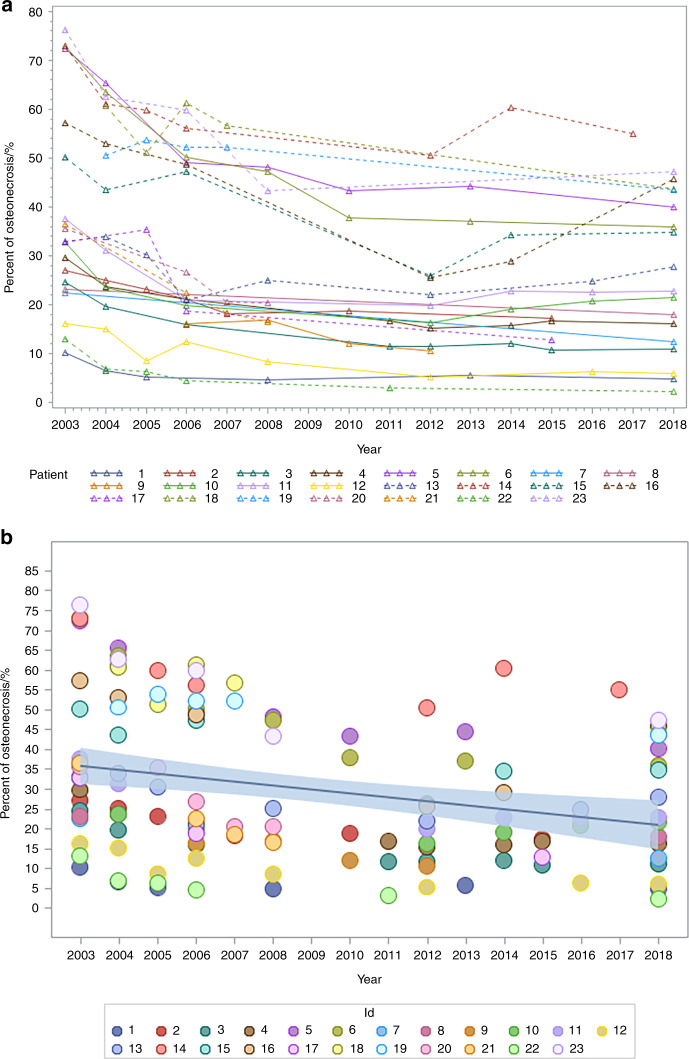
Trends in percentage change of osteonecrotic volume on hip MRI scan. **a** Change in the percentage of osteonecrotic volume. **b** Bubble plots and linear regression of the percentage of osteonecrotic volume

We determined the percentage of osteonecrotic volume by MRI and plotted the results from 2003 to 2018 (Fig. 3a, b).

The linear regression showed that the proportion decreased during the follow-up period (*P* = 0.0011). The mixed-model showed that the percentage of necrotic volume differed each year from 2003 to 2018 (*F* = 5.62, *P* < 0.001). The volume of osteonecrosis decreased rapidly from 2003 (38.83 ± 21.01)% to 2005 (30.38 ± 21.23)% (*P* = 0.000 2), diminished slowly from 2005 to 2013 (28.99 ± 20.59)% and plateaued until 2018 (25.52 ± 15.51)%.

Among the 23 limbs in 15 patients with femoral head necrosis, the ARCO stage of 16 limbs in ten patients stabilized during the follow-up (three in stage IIA, eight in stage IIB, four in stage IIC, and one in stage IIIC). The stage of one limb, however, was downgraded from stage IIB to stage IIA, while the stages of six limbs in four patients were upgraded. In the worsened cohort, the ARCO stage of one limb progressed from stage IIC in 2003 to stage IIIC in 2007 and remained stable in 2018, one patient was upgraded from stage IIC in 2003 to stage IIIC in 2006 and progressed to stage IV in 2012, and one patient worsened from stage IIIC in 2004 to stage IV in 2007 within a short time. In addition, one limb was upgraded from stage IIC in 2003 to stage IIIC in 2006 and then progressed to stage IV in 2008 (Table 3).

**Table 3:** The ARCO stage of fifteen patients with femoral head necrosis and progression

	2003	2004	2005	2006	2007	2008	2009	2010	2011	2012	2013	2014	2015	2016	2017	2018
Case1	IIA	IIA	IIA			IIA					IIA					IIA
Case2	IIB	IIB	IIB	IIB	IIB			IIB					IIB			
Case3-R	IIB	IIB		IIB					IIB	IIB		IIB	IIB			IIB
Case3-L	IIB	IIB		IIB					IIB	IIB		IIB	IIB			IIB
Case4-R	IIC	IIC		IIC		IIC		IIC			IIC					IIC
Case4-L	IIC	IIC		IIC		IIC		IIC			IIC					IIC
Case5-R	IIB															IIB
Case5-L	IIB															IIB
Case6				IIA		IIA		IIA		IIA						
Case7-R	IIC	IIC		IIC						IIC		IIC		IIC		IIC
Case7-L	IIC	IIC		IIC						IIC		IIC		IIC		IIC
Case8-R	IIB	IIB	IIB	IIB		IIB				IIB				IIB		IIB
Case8-L	IIC	IIC	IIC	IIIC		IIIC				IIIC				IIIC		IIIC
Case9	IIIC	IIIC	IIIC	IIIC						IIIC		IIIC			IIIC	
Case10-R	IIC	IIC		IIIC						IV		IV				IV
Case10-L	IIC	IIC		IIIC						IV		IV				IV
Case11	IIB		IIB	IIB									IIA			
Case12-R		IIIC	IIIC	IIIC	IV											IV
Case12-L		IIIC	IIIC	IIIC	IV											IV
Case13-R	IIB			IIB	IIB	IIB										
Case13-L	IIB			IIB	IIB	IIB										
Case14 Case15	IIA IIC	IIA IIC	IIA	IIA IIIC		IV			IIA							IIA IV

In general, the clinical stage showed that femoral head necrosis progressed slightly from 2003 to 2007. Six limbs in four patients showed progressive worsening. The stages reached a plateau from 2007 to 2018, when we detected no progression. One patient improved in clinical stage, and the remaining 16 limbs of ten patients stabilized in clinical stage over the 15 years.

Forty patients underwent joint function assessments in March 2018. The average Harris hip score in patients with osteonecrosis of the femoral head on MRI was 69.69 ± 13.72 (*n* = 13), while the mean score in patients without necrosis was 78.70 ± 11.94 (*n* = 27). There was a significant difference between the two groups (*t* = −2.131, *P* = 0.04). Among the 40 patients, 29 (72.5%) had relatively stable hip joint function during the 15-year follow-up, seven (17.5%) had a mild decline, and four (10%) had severe deterioration.

## Discussion

SARS emerged on November 16, 2002 in Foshan City, Guangdong Province, China, and spread to Southeast Asia and then around the world. Currently, 15 years after the initial treatment of the disease, the biological behavior of the SARS virus and the current status of damaged organs after recovery remain unclear. Lung inflammation was the dominant radiological feature of SARS infection, and high-dose steroid pulse therapy to suppress the unbalanced inflammation caused femoral head necrosis. The dynamic variations in pulmonary imaging findings, pulmonary function and femoral head necrosis have attracted attention from physicians worldwide.^8,9^ Until now, however, there have been no comprehensive studies on the health utility outcomes of SARS patients after a follow-up period of over 5 years. Inadequate personal protective equipments against the disease during the initial outbreak resulted in nosocomial infection among healthcare workers at Peking University People’s Hospital. These patients became infected in identical environments and same geographic area and received similar treatment regimens and rehabilitation plans, constituting a truly unique cohort worldwide for studying the natural course of the SARS infection and steroid-induced femoral head necrosis.

Ruuskanen et al. reported that ~200 million people worldwide develop viral pneumonia each year.^10^ In the past 10 years, apart from SARS coronavirus, avian influenza virus, and Middle East respiratory syndrome coronavirus periodically had outbreaks, and the pulmonary images of viral pneumonia usually presented with multileaf interstitial changes, ground glass opacities and consolidation; however, antibiotics were ineffective for treating these conditions.^11^ Conventional views dictated that viral pneumonia was self-limiting. Das et al. followed up 55 patients with MERS pneumonia, analyzed imaging variations within 1 year after rehabilitation, and found that the most common type of lesion was ground-glass changes in the periphery of the lung.^12^ Chen et al. followed up 56 patients with H7N9 avian influenza to analyze pulmonary function and imaging changes within 2 years after infection.^13^ The results showed that despite interstitial changes and fibrosis on imaging, ventilation and diffusion dysfunction improved to some extent after 6 months; however, recovered patients experienced impaired pulmonary function over those 2 years. In a cohort of 48 H1N1 pneumonia cases, Liu et al. found that patients who showed mild to moderate pulmonary dysfunction manifested with diffusion disorder (33.3%) and small airway dysfunction (33.3%).^14^ Wu et al. at the Beijing Friendship Hospital found that the lesions on CT scans of 11 SARS patients both 6 months and 84 months after rehabilitation clearly diminished compared with those seen at 3 months.^15^ Ground-glass opacity (GGO) lesions were dominant at 6 months, while intralobular and interlobular septum thickening were prominent at 84 months. Dundamadappa et al. acquired high-resolution CT scans of 65 SARS patients at 6 weeks, 3 months, 6 months and 1 year. The initial scan of 28 patients (45.9%) showed normal scarring and fibrosis (parenchyma and stroma).^16^ That study concluded that persistent GGOs may be characterized by fibrosis rather than alveolar inflammation. Ong et al. evaluated the pulmonary function and health utility of 94 patients 1 year after being rehabilitated from SARS.^17^ The outcomes showed that 63% of the patients maintained normal lung function, with slightly reduced FEV1/FVC values in 5% of patients and moderately a reduced FVC value in one case. Of these patients, 18% and 3% had mildly and moderately impaired diffusion function, respectively.

To date, no imaging data of lung injury in SARS patients have been reported internationally with a follow-up period of over 5 years. We conducted a cohort study of 80 healthcare workers who were infected with nosocomial SARS at Peking University People’s Hospital in 2003. With radiology examinations, we distinguished GGO and strip-like consolidation on CT scans and calculated the percentage of areas with different lesions. Our study is the first to describe the recovery curve of lung injury in SARS patients over the past 15 years. We found that the percentage of lesions rapidly decreased from 2003 to 2004 and plateaued from 2004 to 2018. These results suggest that SARS-induced pulmonary lesions visualized with imaging recovered to a greater extent within 1 year after rehabilitation. These results indicated that there was a correlation between pulmonary CT images and functional changes. Thus far, there has been no report on the pulmonary function in SARS patients during a follow-up period of over 2 years on the global scale. Our study showed that the pulmonary function in 2018 was basically the same as that in 2006, with mildly impaired diffusion function. Although there was no substantial recovery, we should take the natural degeneration of pulmonary function over the past 15 years into consideration. In addition, the pulmonary function of patients who had normal findings on CT after recovering from SARS in 2003 was substantially better than that of patients with abnormalities. This implies that pulmonary function could be improved to a larger extent when the acute phase of infectious viral pneumonia is effectively managed. This finding has great value in judging and predicting the prognosis of viral pneumonia.

Previous studies have reported that subchondral osteonecrosis occurred in 5%–10% of patients with SARS according to MRI scans after systemic steroid treatment and that the cumulative steroid dose was a risk factor for osteonecrosis.^18,19^ However, the long-term outcomes of osteonecrosis resulting from high-dose steroids were unavailable. Our study, as far as we know, is the first to report that the status of femoral head necrosis caused by high-dose steroid pulse therapy to treat infectious viral pneumonia could remain stable over the long term and that femoral head necrosis could even become reversible. This phenomena clearly differs from the progression of femoral head necrosis caused by long-term steroid administration for other diseases (e.g., leukemia, nephrotic syndrome, rheumatoid arthritis).^20^ Despite limited weight-bearing protocols and having an improved blood supply, femoral head necrosis induced by long-term steroid administration for leukemia, nephropathy and rheumatoid arthritis was always progressive and irreversible and ultimately led to worsened necrosis and subsequent collapse of the femoral head.^21,22^ Loss of joint function and arthritis were inevitable consequences. ARCO stage II femoral head necrosis progressed to stage III over a follow-up ranging from 2 months to 3 years (average 17 months), collapsed within 3–16 months (average 11 months), and progressed to stage IV with arthritis within 6 years.^23^ Shimuzu et al. reported that it took an average of 33 months to progress from stage I lesions to stage III and 49 months from stage I to stage IV.^24^ Wei et al. reported that steroid-induced femoral head necrosis in stage I progressed to stage IV after 24 months.^25^

In our study, femoral head necrosis occurred in 23 limbs from 15 SARS patients (15/71) and was presumed to be closely related to short-term high-dose steroid therapy and had little direct relation with the SARS-CoV infection. Our study is the first to describe the variation curve of femoral head necrosis of SARS patients over a period of 15 years by measuring the volume on continuous MRI images. The results showed that the volume of osteonecrosis in 15 patients substantially decreased, demonstrating an overall tendency toward improvement. Over the past 15 years, femoral head necrosis in the cohort recovered rapidly between 2003 and 2005, with a slower pace between 2005 and 2012, and reached a plateau from 2012 to 2018. The hip function questionnaire showed that 29 (72.5%) patients remained stable over 15 years, with limited hip function in seven (17.5%) patients, while four (10%) developed arthritis accompanied with poor mobility. These outcomes show that the limited hip joint function progressed slowly. Femoral head necrosis also lessened to some extent, and correspondingly, the patients’ hip joint function was generally stable over the past 15 years.

These results aid in understanding the natural course of pulmonary recovery after viral infection and femoral head necrosis induced by high-dose steroids. These findings have profound implications for physician understanding of clinical steroid therapy.

## Methods

All 78 surviving SARS patients at Peking University People’s Hospital were included in our observational study from August 2003 to March 2018. The patients underwent pulmonary CT scans and hip joint MRI scans annually from 2003 to 2018 and pulmonary function tests in 2006 and 2018. Due to the uniqueness of SARS patients, the decision to undergo physical examinations and imaging entirely depended on the patients’ choices.

We calculated the percentage of area with GGO lesions and strip-like consolidations on chest CT scans and analyzed the variation curve of the proportions. We acquired CT scans by using a GE 64-slice Light Speed CT scanner and GE 256-slice Revolution scanner. A high-resolution multislice spiral CT scan from the apex to the base of the lung was obtained with the patient holding their breath at the end of maximal inspiration. The slice thickness was 10 mm (2003–2005) or 0.625 mm (after 2005). Two physicians with more than 5 years of experience in chest CT review analyzed all chest CT images from disease onset through the follow-up period of 2003–2018. We selected lung window images from the tracheal bifurcation plane and the right diaphragm upper plane and calculated the percentage area of the corresponding lung field layer with ground-glass shadows and cord and interstitial lesions in the two planes. We recorded the percentage of area with the intrapulmonary lesion and analyzed the variation trends.

We performed pulmonary function tests via an automatic pulmonary function testing system (master screen-pft manufactured by Jaeger, Germany) in 2006 and 2018. The main parameters included TLC, residual volume (RV), FVC, FEV_1_, FEV_1_/FVC ratio, and forced expiratory flow at 25%–75% of the pulmonary volume (FEF_25%–75%_). We measured the DLCO using the single-breath method. FEV_1_/FVC <70% was defined as obstructive ventilation dysfunction, TLC <80% of the predicted value indicated restrictive ventilation dysfunction, DLCO <80% of the predicted value indicated reduced lung diffusion capacity, and a value <65% of the predicted value defined impaired FEF_25%–75%_ values.

We calculated the percentage of volume with osteonecrosis in the femoral head and analyzed its variation at various time points. Consecutive coronal T1WI images were sent to a picture archiving and communication system to measure the volume of the femoral head with necrosis. On the basis of the unique feature of femoral head necrosis, we used the serpiginous low signal band as the margin, confirmed the inner side as the necrotic area, and calculated the percentage of volume with osteonecrosis as (necrotic volume/femoral head volume)×100%. We used the ARCO stage classification standard to assess the clinical femoral head necrosis stage.^26^ We also performed a general condition survey and assessed hip joint function with the Harris score in March 2018 via questionnaire.^27^

To avoid potential judgment bias, we ensured that each person was imaged with the same CT and MRI system during follow-up. The standardized image judging, data extraction, data processing, and calculation process were performed by different research groups. Three senior radiologists, two senior orthopedic surgeons, and two senior respiratory physicians confirmed the pathological judgment standard based on imaging. Imaging data analysis was independently performed and averaged by three senior radiologists. Two professional statisticians independently performed the data processing and calculation processes.

This study was registered with ClinicalTrials.gov (NCT03443102) and was authorized by the Ethics Committee of Peking University People’s Hospital (2018PHB010-01). All recruited patients provided written informed consent to participate.

### Statistical methods

We used numbers and percentages to present categorical variables and mean ± SD or median (P_25_, P_75_) to present continuous variables. We used the two-sample *t* test or Satterthwaite *t* test to compare the lung function variables between patients who had abnormal CT and normal CT findings. We use the mixed-model repeated-measures analysis to compare model-adjusted least-squares means over 15 years. The model included the fixed effects of the follow-up years, and the random effect (random intercept) was the “participant”. We performed all statistical analyses using SAS version 9.4 (SAS Institute, Cary, NC), and all testing was two-sided with the significance level set at *P* < 0.05.
