# Correction: Long-term bone and lung consequences associated with hospital-acquired severe acute respiratory syndrome: a 15-year follow-up from a prospective cohort study

**DOI:** 10.1038/s41413-020-00113-1

**Published:** 2020-09-21

**Authors:** Peixun Zhang, Jia Li, Huixin Liu, Na Han, Jiabao Ju, Yuhui Kou, Lei Chen, Mengxi Jiang, Feng Pan, Yali Zheng, Zhancheng Gao, Baoguo Jiang

**Affiliations:** 1 Department of Orthopedics and Trauma, Peking University People’s Hospital, Beijing, China; 2 Department of Respiratory Medicine, Peking University People’s Hospital, Beijing, China; 3 Department of Clinical Epidemiology and Biostatistics, Peking University People’s Hospital, Beijing, China; 4 Department of Central Laboratory, Peking University People’s Hospital, Beijing, China; 5 Department of Education, Peking University People’s Hospital, Beijing, China; 6 Department of Radiology, Peking University People’s Hospital, Beijing, China

**Keywords:** Calcium and phosphate metabolic disorders, Bone

Correction to: *Bone Research* 10.1038/s41413-020-0084-5, published online 14 February 2020

During a re-read of our article^1^ previously published in Bone Research, we regrettably found a omitting in the acknowledgement section relating to the supporting foundation.

That work was also supported by the Key Laboratory of Trauma & Neural Regeneration (Peking University), Chinese Ministry of Education (Number: BMU2019XY007-01); and Major R&D Program of Chinese National Ministry of Science and Technology (2018YFB1105504).
